# Optimize the farming system to improve the physical and chemical properties of soil in Northeast China, thereby increasing maize yield

**DOI:** 10.3389/fpls.2025.1626882

**Published:** 2025-08-11

**Authors:** Xiangkun Qi, Weidong Huang, Yicong Li, Jiachuang Xie, Fenglin Huang, Yufeng Wang, Jian Fu, Kejun Yang

**Affiliations:** College of Agronomy, Heilongjiang Bayi Agricultural University, Key Laboratory of Modern Agricultural Cultivation and Crop Germplasm Improvement of Heilongjiang Province, Daqing, China

**Keywords:** maize, tillage methods, soil nutrients, aggregate, production

## Abstract

Northeast China’s black soil region faces soil fertility decline, inadequate straw usage, and low maize yields. To address these issues, we conducted a two-year field experiment. The seven treatments comprised rotary ridge tillage (Con), no-tillage (T1), straw return + no-tillage (T2), deep-plowing straw return + ridge tillage (T3), deep-plowing straw return + flat tillage (T4), straw crushing and return + ridge tillage (T5), and straw crushing and return + flat tillage (T6). We examined the impact of various tillage methods on the structure of soil water-stable aggregates, soil nutrients, enzyme activity, and maize yield. The findings indicated that from 2021 to 2022, the soil macroaggregate content in the T4 considerably increased by 23.52% compared to the Con. Compared to Con, T4 significantly increased the mean weight diameter (MWD) and geometric mean diameter (GMD), enhancing soil fertility. Additionally, T4 reduced bald tip length while boosting the 100-Kernels weight by 24.01%, ultimately increasing maize yield by 13.62%. Consequently, deep-plowing straw return + flat tillage significantly enhanced soil structure, augmented soil fertility, and elevated maize production, rendering it the most appropriate tillage strategy for this region.

## 1 Introduction

Maize is among the most essential food crops globally. As the world’s second largest producer of maize. China constitutes 22.4% of global maize production while employing under 20% of the total worldwide maize growing area (FAOSTAT, 2021). The semi-arid area in western Heilongjiang Province is a principal maize production zone in China, where ridge tillage is the primary spring cultivation method. Prolonged agricultural automation and constant tillage have resulted in soil structure deterioration and a loss in fertility (Afshar et al., 2022). The region faces arid springs and wet summers, leading to severe soil moisture depletion under conventional ridge tillage in spring and waterlogging risks in summer. These conditions significantly reduce maize production in the area. Consequently, examining suitable agricultural practices in this region is essential for guaranteeing food security and safeguarding the agro-ecological ecosystem. Different tillage systems can significantly alter soil properties, including physical, chemical, and biological characteristics. Conventional tillage methods often caused substantial soil erosion and water depletion, ultimately resulting in decreased soil fertility and degradation of the agro-ecological environment (Huang et al., 2018). In contrast to conventional tillage methods, no-tillage increased soil bulk density, which could inhibit crop root growth and development (Ji et al., 2013). Flat tillage methods could markedly decrease soil water evapotranspiration and improve soil moisture retention capability (Liang et al., 2021). Straw incorporation was widely acknowledged as an essential agricultural method for improving soil fertility (Zhao et al., 2019). Straw mulching has been shown to increase soil organic matter content (Akhtar et al., 2018), significantly reduce soil moisture evaporation, mitigate soil compaction, and diminish soil nutrient loss (Lv et al., 2023). Deep plowing straw returning could significantly mitigate soil compaction and adhesion, reduce insect and disease prevalence, facilitate straw decomposition, and improve soil fertility (Al-Kaisi et al., 2014; Liu et al., 2022). Therefore, when analyzing the impact of tillage systems on soil physicochemical characteristics, it is essential to consider the incorporation of diverse straw return schemes with tillage practices.

China’s semi-arid western regions face three critical challenges: deteriorating soil fertility, underutilized straw resources, and suboptimal maize productivity. Implementing conservation tillage with straw return methods presents an effective solution to these problems (Chen et al., 2022; Zhao et al., 2023). The efficacy of conservation tillage and straw return strategies for soil enhancement varies considerably across diverse regions and environmental situations. We conducted a two-year field experiment to evaluate different tillage and straw returning methods on soil aggregate stability, nutrient dynamics, enzyme activity, and maize yield. We hypothesized that deep-plowing straw return + flat tillage improved soil aggregate stability, enhanced fertility, and increased yield, making it the optimal tillage method for this region. This research establishes a theoretical basis for optimizing tillage methods, improving straw resource management, and attaining elevated maize yields in the semi-arid areas of western Heilongjiang Province.

## 2 Materials and methods

### 2.1 Experimental site

The research location is situated in Zhao Zhou County, Heilongjiang Province (N 46°00′28″, E 125°3 2′81″). The area is level, characterized by an average frost-free duration of 130–135 days. **Supplementary Figure S1** illustrates the air temperature and precipitation distributions for the maize growing seasons of 2021 and 2022. The soil is classified as chernozem. The fundamental fertility parameters of the topsoil at a depth of 0–20 cm are as follows: organic carbon, 14.83 g kg^-1^; pH, 7.9; total nitrogen, 1.19 g kg^-1^; available phosphorus, 17.2 mg kg^-1^; and available potassium, 240.4 mg kg^-1^.

### 2.2 Experimental design

This experiment included a positioning assessment, with data collected annually from 2021 to 2022. In this paper, positioning research denotes the process of ongoing investigation, observation, and analysis conducted at a designated site, maintaining both the testing location and agricultural practices throughout the study. Seven treatments were employed for comparison in the experiment: rotary ridge tillage (Con), no-tillage (T1), straw return + no-tillage (T2), deep-plowing straw return + ridge tillage (T3), deep-plowing straw return + flat tillage (T4), straw crushing and return + ridge tillage (T5), and straw crushing and return + flat tillage (T6) (**Table 1**). Rotary ridge tillage was used as a Con. The tested variety is ‘Dongxu 20’. Each treatment was repeated three times with a random block arrangement, and the experimental plot was designed to be 0.33 hm^2^ (100 m×32.5 m). The planting density of the maize was 75000 plants hm^-2^, and the compound fertilizer was applied at 650 kg hm^-2^ (N:P_2_O_5_:K_2_O = 27:10:12). All the farming practices including herbicide and insecticides were performed in each plot and consistent with local agronomic practices during the entire experimental seasons. Maize was planted on 2 May 2021 and 28 April 2022 and harvested on 1 October 2021 and 2 October 2022.

**Table 1:** Experimental design.

Treatment	Measure
Rotary ridge tillage (Con)	After mechanical harvesting in autumn, all straw was removed from the field. In spring, rotary tillage and ridging were conducted at a depth of 15–20 cm, followed by sowing and a one-time application of base fertilizer into the 15–20 cm soil layer.
No-tillage (T1)	After mechanical harvesting in autumn, all straw was removed from the field. In spring, no-tillage practices were implemented, followed by flat sowing of maize and a one-time application of base fertilizer into the 15–20 cm soil layer.
Straw returning + no-tillage (T2)	After autumn harvest, all straw was crushed to sh0 cm lengths and evenly distributed as surface mulch. In spring, no-tillage practices were implemented, followed by flat sowing of maize and a one-time application of base fertilizer into the 15–20 cm soil layer.
Deep plowing straw returning + ridge tillage (T3)	After mechanical harvesting in autumn, all straw was crushed to sh0 cm lengths and the straw was deep plowed into the field to a depth of 25–30 cm. In spring, rotary tillage and ridging were conducted, followed by sowing and a one-time application of base fertilizer into the 15–20 cm soil layer.
Deep plowing straw returning + flat tillage (T4)	After mechanical harvesting in autumn, all straw was crushed to sh0 cm lengths and the straw was deep plowed into the field to a depth of 25–30 cm. Without ridging, sowing was conducted in spring, accompanied by a one-time application of base fertilizer into the 15–20 cm soil layer.
Straw crushing and returning + ridge tillage (T5)	After mechanical harvesting in autumn, all straw was crushed to sh0 cm lengths and evenly distributed on the soil surface. The straw was then incorporated into the 0–20 cm soil layer using a combined soil preparation machine. In spring, rotary tillage and ridging were conducted, followed by sowing and a one-time application of base fertilizer into the 15–20 cm soil layer.
Straw crushing and returning + flat tillage (T6)	After mechanical harvesting in autumn, all straw was crushed to sh0 cm lengths and evenly distributed on the soil surface. The straw was then incorporated into the 0–20 cm soil layer using a combined soil preparation machine. Without ridging, sowing was conducted in spring, accompanied by a one-time application of base fertilizer into the 15–20 cm soil layer.

### 2.3 Sample collection

Soil samples were taken at depths of 0–10 cm, 10–20 cm, and 20–30 cm using a soil auger during the maize jointing, tasseling, and maturity phases throughout the 2021–2022 growth seasons for each treatment. Residual roots, straw pieces, and other contaminants were manually extracted from the soil samples, which were subsequently brought to the laboratory in sealed containers for drying, grinding, and sifting prior to analysis. Soil water-stable aggregates were categorized by the wet sieving technique (Elliott, 1986), yielding six specific size classifications: >5 mm, 2–5 mm, 1–2 mm, 0.5–1 mm, 0.25 - 0.5 mm, and<0.25 mm.

### 2.4 Measurement and methods

#### 2.4.1 Measurement of the soil nutrient content

The soil organic matter content was determined via the potassium dichromate volumetric method (Nelson, 1982). The soil total nitrogen content was determined via digestion with H_2_SO_4_ and an automatic Kjeldahl apparatus (Nelson and Sommers, 1980). The soil-available nitrogen was determined via the sodium hydroxide-boric acid-available nitrogen diffusion method (Mulvaney and Khan, 2001). Soil-available phosphorus was extracted via the 0.5 mol L^-1^ NaHCO_3_ colorimetric method (Truog, 1930). Soil-available potassium was measured via atomic absorption spectrophotometry with 0.5 mol L^-1^ NH_4_OAc (Mehlich, 2008).

#### 2.4.2 Measurement of soil enzyme activity

1. The catalase activity was determined based on the method described by (Ren et al., 2016). Two grams of each air-dried soil sample was weighed and placed in a 100 ml conical bottle, 5 mL of 3% H_2_O_2_ solution and 40 mL of deionized water were added, and the mixture was shaken for 20 min and then filtered. Five milliliters of H_2_SO_4_ solution containing 1.5 mol L^-1^ H_2_SO_4_was added to the clear liquid, and then 25 ml of H_2_SO_4_ solution was removed after filtration. The volume of KMnO_4_ solution consumed was recorded by titrating with 0.02 mol L^-1^ KMnO_4_ solution until the liquid was slightly red and did not change color for 30 s.
2. The urease activity was determined based on the method described by Ren et al. (2016). Fresh soil samples (5 g) were transferred to a 100-ml conical flask and were incubated by adding citrate solution (20 ml, pH 6.7) and urea solution (10 ml, 10%, w/v) for 24 h at 37°C. The mixed solution was shaken at 180 rpm for 20 min and then filtered. Then, the filtrate (1 ml), sodium hypochlorite solution (3 ml), and so­dium phenol solution (4 ml) were mixed in a 50-ml volumetric flask. Deionized water was added to the volumetric flask to a constant volume of 50 ml. The absorbance of the solution was measured at 578 nm using a spectrophotometer (UV2550, Shimadzu, Japan).
3. The alkaline phosphatase activity was determined based on the method described by Ren et al. (2016). Five grams of air-dried soil was placed in a 200-triangle bottle with 2.5 ml of toluene. After 15 min, 20 ml of 0.5% sodium phenyldisodium phosphate was added, the reaction mixture was incubated at 37°C, the mixture was cultured for 24 h, 100 ml of 0.3% aluminum sulfate solution was added to the culture medium, and the mixture was filtered with dense filter paper. Three milliliters of filtrate was placed into a 50 ml volumetric bottle, 5 ml of buffer and 4 drops of chloroform-p-benzoquinone imide reagent were added to each bottle, the mixture was diluted to scale after color development, and the color at a wavelength of 660 nm was compared via a spectrophotometer.

#### 2.4.3 Evaluation of soil aggregate stability indices

(1) The mass percentage of aggregates at a given particle size level was calculated according to the method described by (Choudhury et al., 2014)


$${\text{W}}_{i}=\frac{M_{i}}{M}\times 100\%$$

where *M_i_* is the mass of the i-level soil water-stable aggregate after sieving (g), and *M* was applied to determine the total mass of the aggregates (g).

(2) Soil aggregate stability was determined using the MWD and GMD. The calculation formulas of MWD and GMD (Haynes and Swift, 1990) are as follows:


$$\text{MWD}=\left[\frac{\sum_{i=1}^{n}\left(W_{i}X_{i}\right)}{\sum_{i=1}^{n}W_{i}}\right]$$

$$\text{GMD}=\text{exp}\left[\frac{\sum_{i=1}^{n}\left(W_{i}\ln X_{i}\right)}{\sum_{i=1}^{n}W_{i}}\right]$$

where *W_i_* is the weight percentage of each aggregate (%), and *X_i_* is the average diameter (mm) of each particle size.

(3) The fractal dimension of the soil water-stable aggregates was calculated following (Rasiah et al., 1993)


$$\text{D}=3-\text{lg}\left[\frac{\text{M}(\text{r}<{\text{X}}_{\text{i}})}{{\text{M}}_{\text{T}}}]/\text{lg}[\frac{{\text{X}}_{\text{i}}}{{\text{X}}_{\text{max}}}\right]$$

where M (r< X_i_) is the aggregate weight (g) with a particle size smaller than X_i_, M_T_ is the total weight of the aggregates, X_i_ is the average diameter of a certain level of the aggregate, and X _max_ is the maximum particle size of the aggregates.

#### 2.4.4 Measurement of maize yield

At the maize maturity stage, four rows (5 m long with 0.65 m row spacing) were selected from the central area of each plot. All maize ears were harvested, and the grain moisture content was measured using a PM8818 moisture analyzer. The final yield was adjusted to a standard moisture content of 14%.

### 2.5 Statistical analysis

Excel 2010 was used to organize the data. Different treatments were compared via Duncan’s test at the 0.05 probability level (P ≤ 0.05). Analysis of variance was performed for grain yield, soil aggregates, organic carbon, soil nitrogen, and soil enzyme activity via SPSS22.0 (SPSS Inc., Chicago, USA). Origin 2021 software (Origin Lab, USA) was used for figure preparation.

## 3 Results

### 3.1 Soil water - stable aggregates

**Figure 1:**
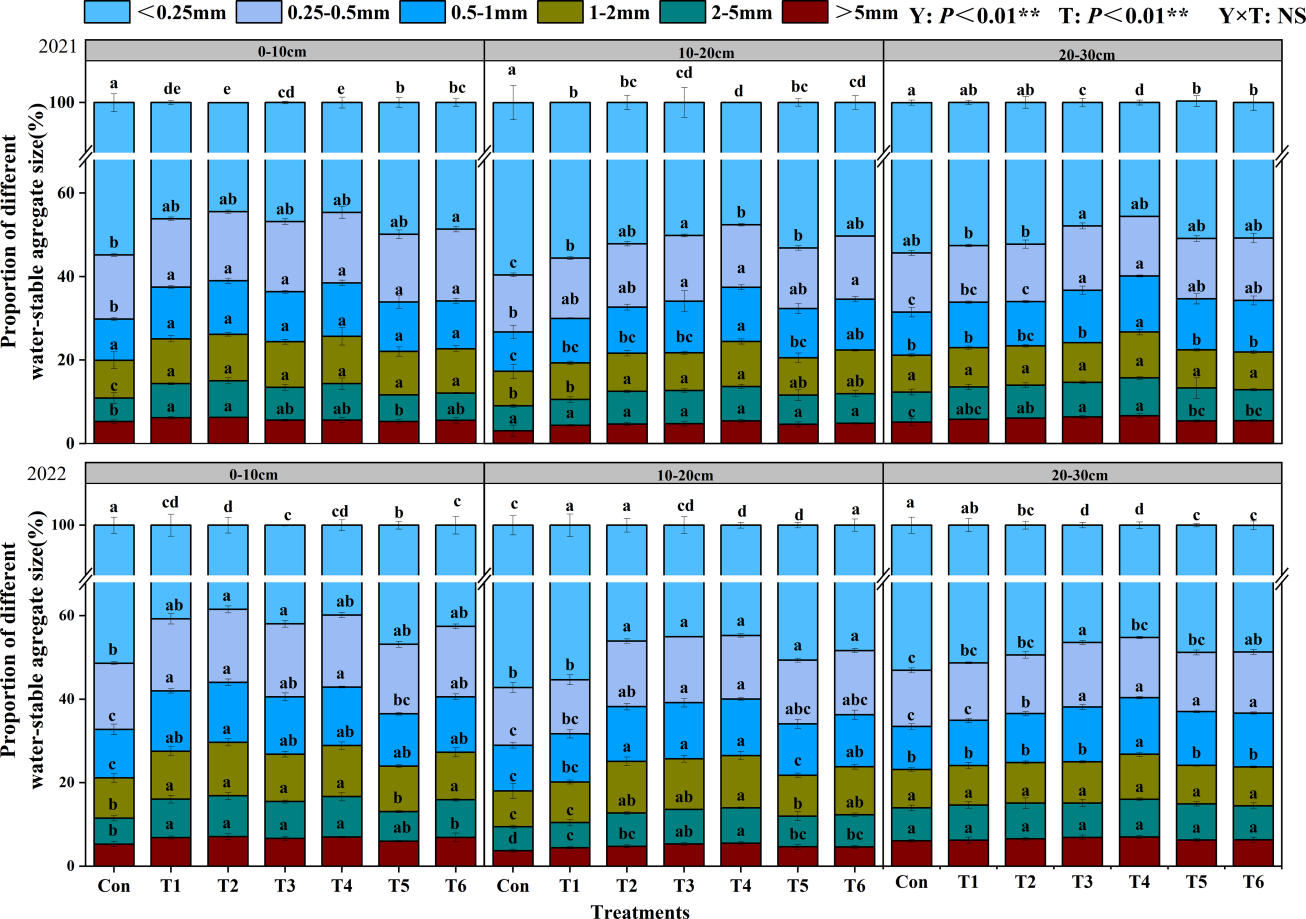
Effects of tillage methods on the particle size composition of soil water-stable aggregates. Con (rotary ridge tillage), T1 (no-tillage), T2 (raw return + no-tillage), T3 (deep plow straw return + ridge tillage), T4 (deep plow straw return + flat tillage), T5 (raw crushing and return + ridge tillage), and T6 (raw crushing and return + flat tillage). Different alphabets indicate the significance within the same year at 5% level by Duncan' tests. In ANOVA, Y, T represent the variable year, treatment. NS, not significant, (p > 0.05). **, Significant at p < 0.01.

Y and T had significant effects on soil aggregate content(P<0.01; **Figure 1**). In 2021 and 2022, the content of soil water-stable aggregates gradually increased as the aggregate particle size decreased. Compared to the Con treatment, all other treatments increased the content of large aggregates. In the 0–10 cm soil layer, all other treatments significantly increased the content of large aggregates compared to the Con treatment. In the 10–20 cm soil layer, the T4 treatment showed the highest contents of water-stable aggregates in the size fractions of > 5 mm, 2–5 mm, 1–2 mm, and 0.5–1 mm, with increases of 54.88%, 45.04%, 42.16%, and 27.01%, respectively, compared to the Con treatment. In the 20–30 cm soil layer, the T4 treatment exhibited the highest content of R > 0.25mm.

### 3.2 Soil water - stable aggregate stability

**Figure 2:**
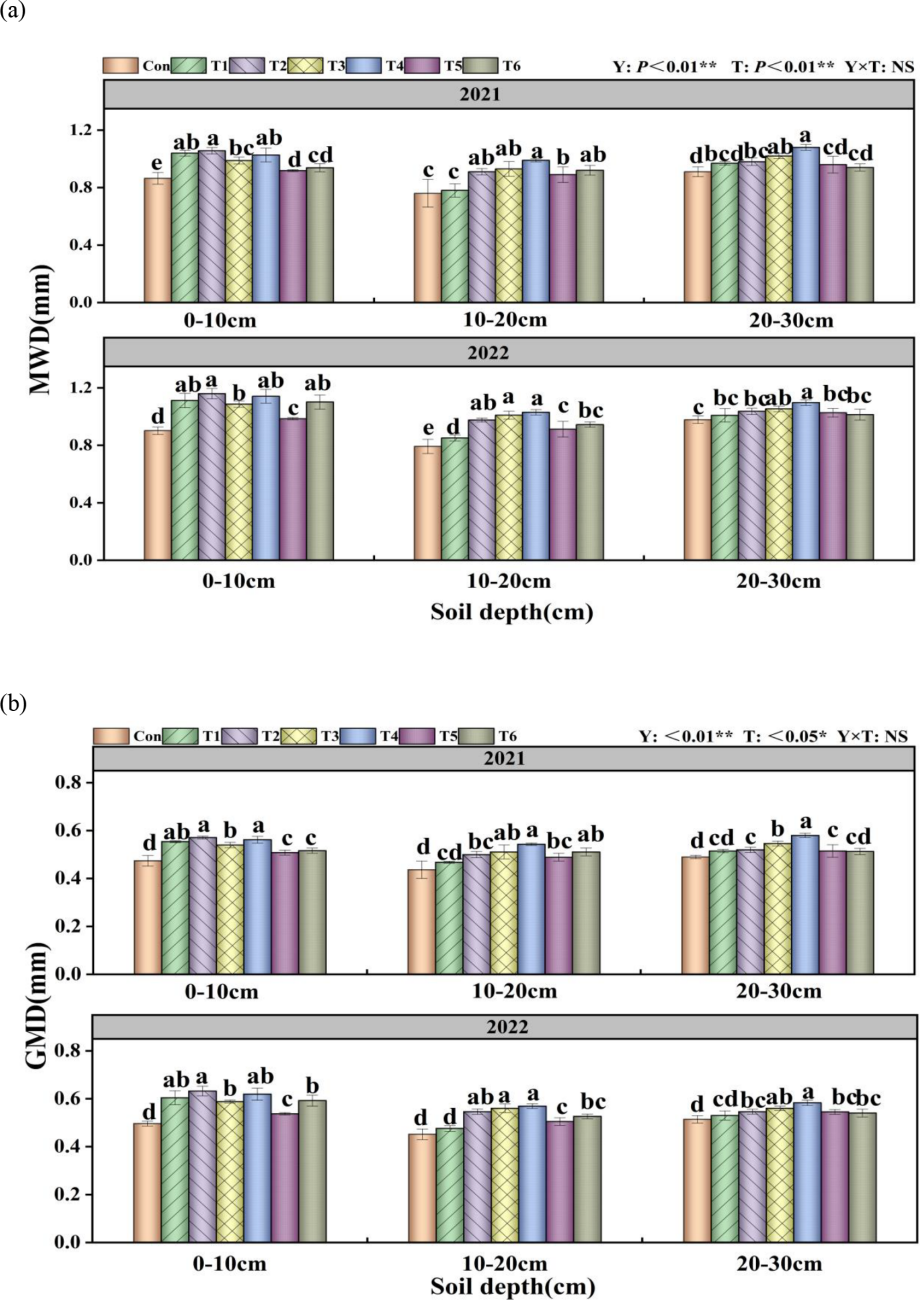
Effects of tillage methods on soil water-stable aggregate stability. Mean weight diameter (MWD) **(a)** and geometric mean diameter (GMD) **(b)**. Con (rotary ridge tillage), T1 (no-tillage), T2 (raw return + no-tillage), T3 (deep plow straw return + ridge tillage), T4 (deep plow straw return + flat tillage), T5 (raw crushing and return + ridge tillage), and T6 (raw crushing and return + flat tillage). MWD, mean weight diameter; GMD, geometric mean diameter. Different alphabets indicate the significance within the same year at 5% level by Duncan' tests. In ANOVA, Y, T represent the variable year, treatment. NS, not significant, (p > 0.05). *, Significant at p < 0.05. **, Significant at p < 0.01.

Y and T significantly influenced MWD (P< 0.01), while T significantly affected GMD (P< 0.01), and T also had a notable impact on GMD (P< 0.05; **Figures 2a, b**). In the 0–10 cm soil layer, the T2 treatment showed the highest MWD and GMD, with significant increases of 25.42% and 23.91%, respectively, compared to the Con treatment. In the 10–20 cm soil layer, all treatments except T1 significantly increased the MWD and GMD of aggregates compared to the Con treatment. In the 20–30 cm soil layer, the T4 treatment exhibited the highest MWD and GMD values, with significant increases of 15.72% and 15.89%, respectively, compared to the Con treatment.

### 3.3 Organic carbon in soil water - stable aggregate

**Figure 3:**
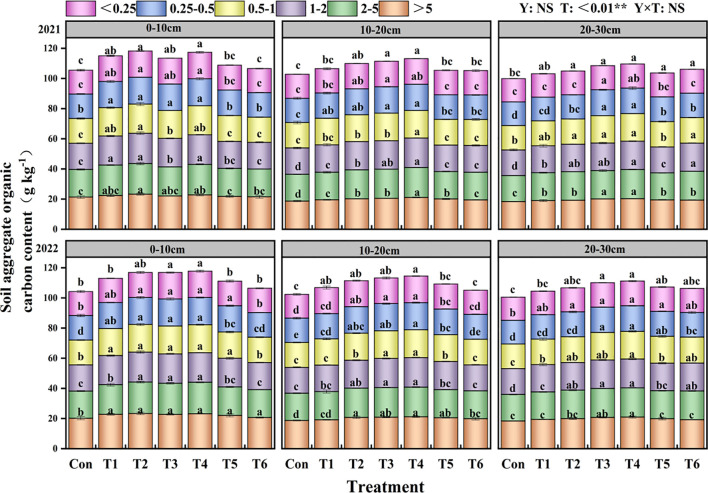
Effects of tillage methods on the soil soil water-stable aggregate organic carbon content. Con (rotary ridge tillage), T1 (no-tillage), T2 (raw return + no-tillage), T3 (deep plow straw return + ridge tillage), T4 (deep plow straw return + flat tillage), T5 (raw crushing and return + ridge tillage), and T6 (raw crushing and return + flat tillage). Different alphabets indicate the significance within the same year at 5% level by Duncan' tests. In ANOVA, Y, T represent the variable year, treatment. NS, not significant, (p > 0.05). **, Significant at p < 0.01.

T significantly influences the organic carbon content of aggregates (P< 0.01; **Figure 3**). In the 0–10 cm soil layer, all treatments significantly increased the water-stable organic carbon content in soil aggregates compared to the Con treatment. In the 10–20 cm soil layer, the organic carbon content of aggregates larger than 5 mm, between 2–5 mm, and between 1–2 mm in the T4 treatment was considerably elevated compared to the Con treatment, with increases of 12.96%, 10.43%, and 12.83%, respectively. Within the 20–30 cm soil layer, the T4 treatment had the greatest organic carbon concentration across all aggregate size fractions.

### 3.4 Soil organic carbon content

**Figure 4:**
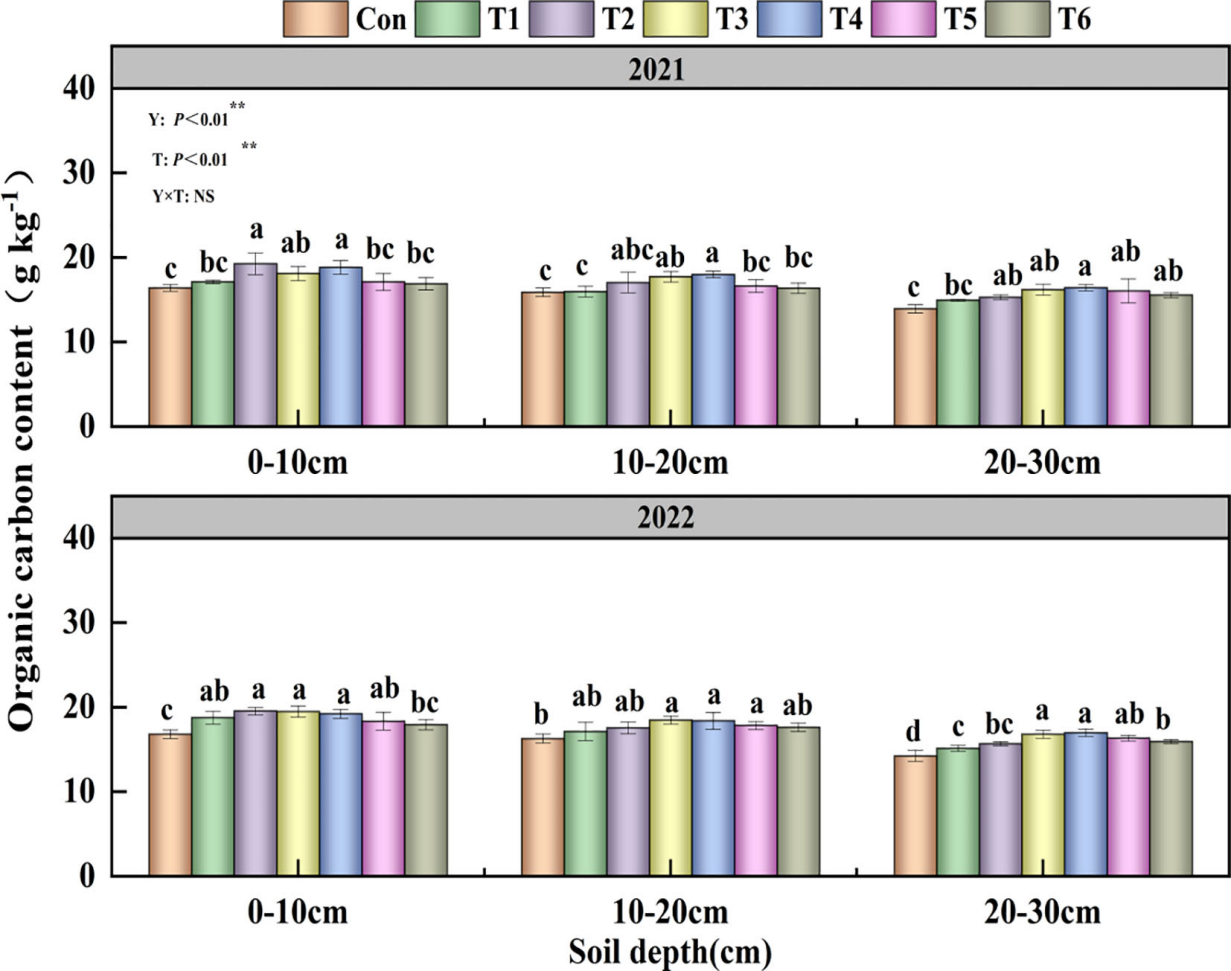
Effects of tillage methods on the soil total organic carbon content. Con (rotary ridge tillage), T1 (no-tillage), T2 (raw return + no-tillage), T3 (deep plow straw return + ridge tillage), T4 (deep plow straw return + flat tillage), T5 (raw crushing and return + ridge tillage), and T6 (raw crushing and return + flat tillage). SOC, soil organic carbon. Different alphabets indicate the significance within the same year at 5% level by Duncan' tests. In ANOVA, Y, T represent the variable year, treatment. NS, not significant, (p > 0.05). **, Significant at p < 0.01.

The influence of Y and T on the soil organic carbon content was highly significant (P<0.01; **Figure 4**). In 2021 and 2022, the SOC content across all treatments shown a progressive decline with increasing soil depth. In the 0–10 cm soil layer, T2 treatment demonstrated a substantial increase of 16.77% relative to the Con treatment. In the 10–20 cm soil layer, the SOC content of the treatments ranked as follows: T4 > T3 > T2 > T5 > T6 > T1 > Con. The T4 treatment exhibited a notable increase of 18.45% in the 20–30 cm soil layer compared to the Con treatment.

### 3.5 Soil nitrogen content

In the 0–10 cm soil layer, the T2 treatment exhibited the greatest TN content during the growth phases from jointing to tasseling (**Table 2**). In the 10–20 cm soil layer, the T4 treatment demonstrated the most significant improvement in TN content, with increases of 15.58%, 16.36%, and 15.26%, respectively, compared to the Con treatment. In the 20–30 cm soil layer, the T3 and T4 treatments shown elevated TN content relative to the Con treatment.

**Table 2:** Effects of tillage methods on the soil total nitrogen content.

Soil depth (cm)	Treatments	Soil total nitrogen content (g kg^-1^)					
		2021			2022		
		Jointing	Tasseling	Maturity	Jointing	Tasseling	Maturity
0~10	Con	1.20c	1.11c	1.20a	1.22c	1.13c	1.22b
	T1	1.32ab	1.13c	1.32a	1.39ab	1.17bc	1.33ab
	T2	1.39a	1.35a	1.37a	1.43a	1.35a	1.39ab
	T3	1.33ab	1.28abc	1.30a	1.37ab	1.30ab	1.41a
	T4	1.37a	1.32ab	1.35a	1.41a	1.34a	1.43a
	T5	1.28bc	1.22bc	1.28a	1.32abc	1.24abc	1.32ab
	T6	1.26bc	1.15c	1.24a	1.28bc	1.19bc	1.26ab
10~20	Con	1.13b	1.09c	1.17b	1.19c	1.11d	1.19c
	T1	1.20ab	1.11c	1.20ab	1.22bc	1.15cd	1.22bc
	T2	1.30a	1.24a	1.30ab	1.32ab	1.24abc	1.33ab
	T3	1.28a	1.24a	1.28ab	1.33ab	1.26ab	1.35ab
	T4	1.33a	1.26a	1.32a	1.35a	1.30a	1.37a
	T5	1.26ab	1.20ab	1.24ab	1.28ab	1.24abc	1.30abc
	T6	1.24ab	1.13bc	1.22ab	1.26abc	1.19bcd	1.28abc
20~30	Con	0.70c	0.68d	0.66c	0.77c	0.72b	0.79c
	T1	0.72bc	0.70cd	0.74abc	0.79c	0.72b	0.83bc
	T2	0.77abc	0.73bcd	0.76abc	0.81c	0.76b	0.92abc
	T3	0.87a	0.83ab	0.96a	0.96ab	0.91a	0.98ab
	T4	0.91a	0.89a	0.92ab	0.98a	0.92a	1.04a
	T5	0.85ab	0.79abc	0.79abc	0.87bc	0.81b	0.96ab
	T6	0.81abc	0.77bcd	0.72bc	0.83c	0.79b	0.94abc
ANOVA							
Y: NS							
T: NS							
Y×T: NS							

Con, rotary ridge tillage; T1, no-tillage; T2, straw return + no-tillage; T3, deep plow straw return + ridge tillage; T4, deep plow straw return + flat tillage; T5, straw crushing and return + ridge tillage; T6, straw crushing and return + flat tillage. TN, total nitrogen; AN, available nitrogen; Different alphabets indicate the significance within the same year at 5% level by Duncan’ tests. In ANOVA, Y, T represent the variable year, treatment. NS, not significant, (p > 0.05). *Significant at p< 0.05. **Significant at p< 0.01.

### 3.6 Soil available phosphorus content

T and Y exerted substantial impacts on AP (P<0.01; **Table 3**). The AP content of the T2 treatment in the 0–10 cm soil layer was superior to that of the T1 treatment. In the 10–20 cm soil layer, the T4 treatment had a markedly higher AP content than the T6 treatment, with increases of 10.81%, 13.92%, and 27.27% from the jointing stage to the maturity stage. In the 20–30 cm soil layer, the T4 treatment exhibited a notable increase in AP content relative to the Con treatment during all growth stages, with increments of 23.54%, 29.88%, and 36.24%, respectively.

**Table 3:** Effects of tillage methods on the soil available phosphorus content.

Soil depth (cm)	Treatments	Soil available phosphorus content (mg kg^-1^)					
		2021			2022		
		Jointing	Tasseling	Maturity	Jointing	Tasseling	Maturity
0~10	Con	32.73c	24.00d	12.74c	33.33d	25.02d	14.24c
	T1	35.07bc	27.25c	18.28a	35.19c	30.08b	18.46ab
	T2	38.20a	31.58a	18.94a	39.77a	33.87a	19.36a
	T3	36.94ab	28.99bc	17.25ab	37.60b	29.66b	17.80ab
	T4	37.60a	30.80ab	18.76a	38.81ab	32.36a	19.06a
	T5	35.01bc	24.78d	14.60bc	35.80c	27.43c	17.01ab
	T6	34.71bc	24.48d	14.00c	35.43c	26.34cd	16.05bc
10~20	Con	27.61d	22.37b	12.62b	28.57d	24.24c	14.12c
	T1	28.51d	22.97b	12.74b	28.75d	24.84bc	14.72bc
	T2	35.25ab	26.04a	17.13a	35.56b	26.16b	17.31a
	T3	36.28a	26.83a	16.65a	37.00a	28.21a	17.37a
	T4	36.40a	27.55a	17.97a	37.60a	28.09a	18.04a
	T5	33.63bc	23.87b	14.42b	33.99c	27.97a	15.51bc
	T6	33.27c	23.21b	13.94b	33.51c	25.74bc	14.36bc
20~30	Con	26.65c	20.26c	11.72d	27.25c	21.77d	12.08d
	T1	27.67c	20.44c	12.20cd	28.03c	21.83d	12.56cd
	T2	31.58ab	22.07b	15.33a	31.82ab	23.94c	15.39ab
	T3	33.03a	25.74a	16.05a	33.51a	27.43ab	16.35a
	T4	32.84a	26.89a	15.75a	33.75a	27.73a	16.65a
	T5	32.61a	23.27b	13.88b	32.85ab	26.52b	14.79b
	T6	30.14b	22.37b	13.34bc	30.98b	24.48c	13.88bc
ANOVA							
Y: **							
T: **							
Y×T: NS							

Con, rotary ridge tillage; T1, no-tillage; T2, straw return + no-tillage; T3, deep plow straw return + ridge tillage; T4, deep plow straw return + flat tillage; T5, straw crushing and return + ridge tillage; T6, straw crushing and return + flat tillage. AP, available phosphorus. Different alphabets indicate the significance within the same year at 5% level by Duncan’ tests. In ANOVA, Y, T represent the variable year, treatment. NS, not significant, (p > 0.05). **Significant at p< 0.01.

### 3.7 Soil available potassium content

T and Y exerted substantial impacts on AK (P<0.01; **Table 4**). In the 0–10 cm soil layer, T2 treatments enhanced the AK content at every growth stage, achieving a statistically significant difference at maturity, with a 14.76% increase relative to the Con treatment. The T4 treatment demonstrated significantly elevated AK content in the 10–20 cm soil layer across all growth stages, with increases of 11.21%, 15.83%, and 12.09%, respectively, compared to the Con treatment. In the 20–30 cm soil layer, the average AK content across treatments exhibited the following descending order: T4 > T3 > T5 > T6 > T2 > T1 > Con.

**Table 4:** Effects of tillage methods on the soil available potassium content.

Soil depth (cm)	Treatments	Soil available potassium content (mg kg^-1^)					
		2021			2022		
		Jointing	Tasseling	Maturity	Jointing	Tasseling	Maturity
0~10	Con	283.25a	237.98a	269.42c	294.37a	236.56b	275.34b
	T1	292.94a	257.09a	272.41bc	300.86a	263.72ab	276.26b
	T2	312.62a	277.33a	307.41a	323.74a	286.60a	317.39a
	T3	299.29a	265.21a	299.57ab	312.48a	272.91ab	312.76a
	T4	307.27a	269.13a	305.99a	319.53a	282.25ab	313.62a
	T5	293.01a	253.95a	296.51abc	309.20a	266.92ab	300.14ab
	T6	298.65a	248.25a	276.40bc	306.70a	264.29ab	282.53b
10~20	Con	270.35c	230.92b	265.14b	280.47a	234.84b	270.63b
	T1	272.9bc	237.62ab	268.42b	281.39a	247.60ab	273.20ab
	T2	290.66ab	248.10ab	282.75ab	307.13a	257.66ab	296.65ab
	T3	293.16a	255.87a	295.87a	309.12a	270.27ab	298.43ab
	T4	298.72a	259.44a	297.65a	313.90a	280.18a	302.85a
	T5	286.53abc	242.04ab	287.03ab	308.20a	262.65ab	291.02ab
	T6	283.75abc	240.98ab	269.28b	305.28a	255.80ab	276.48ab
20~30	Con	254.02b	223.72a	255.87a	261.29a	229.43b	258.58c
	T1	257.37ab	230.00a	261.43a	262.00a	241.19ab	262.15bc
	T2	254.80ab	234.70a	257.37a	259.63a	247.89ab	264.57bc
	T3	275.76a	246.25a	271.27a	280.97a	268.99a	277.69ab
	T4	274.34ab	245.75a	275.91a	282.82a	270.42a	288.81a
	T5	268.78ab	224.44a	265.43a	274.34a	261.72ab	270.77bc
	T6	267.21ab	238.91a	262.08a	271.13a	249.10ab	266.64bc
ANOVA							
Y: **							
T: **							
Y×T: NS							

Con, rotary ridge tillage; T1, no-tillage; T2, straw return + no-tillage; T3, deep plow straw return + ridge tillage; T4, deep plow straw return + flat tillage; T5, straw crushing and return + ridge tillage; T6, straw crushing and return + flat tillage. AK, available potassium; Different alphabets indicate the significance within the same year at 5% level by Duncan’ tests. In ANOVA, Y, T represent the variable year, treatment. NS, not significant, (p > 0.05). **Significant at p< 0.01.

### 3.8 Soil alkaline phosphatase activity

**Figure 5:**
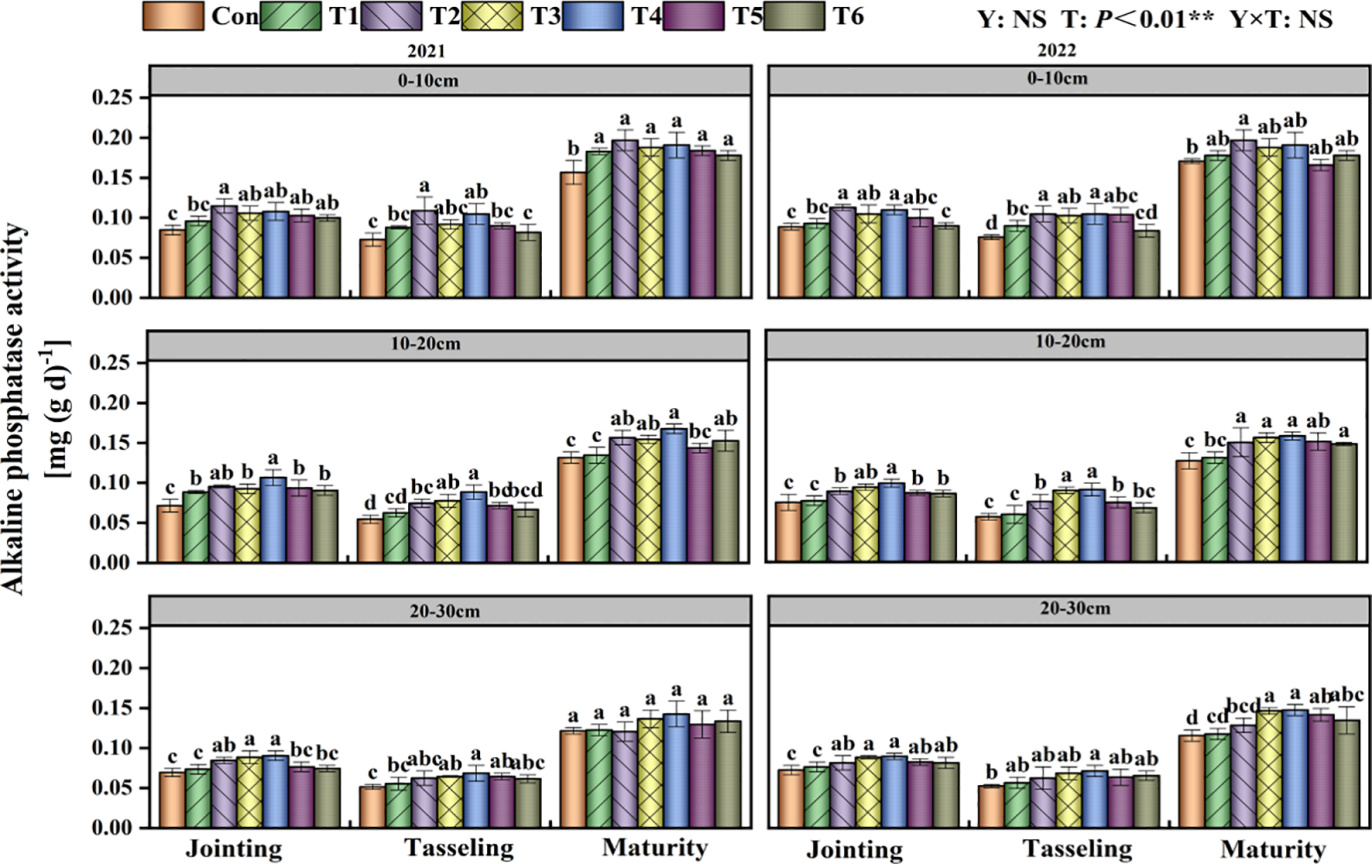
Effects of tillage methods on soil alkaline phosphatase activity. Con (rotary ridge tillage), T1 (no-tillage), T2 (raw return + no-tillage), T3 (deep plow straw return + ridge tillage), T4 (deep plow straw return + flat tillage), T5 (raw crushing and return + ridge tillage), and T6 (raw crushing and return + flat tillage). Different alphabets indicate the significance within the same year at 5% level by Duncan' tests. In ANOVA, Y, T represent the variable year, treatment. NS, not significant, (p > 0.05). **, Significant at p < 0.01.

T had a significant impact on soil alkaline phosphatase activity (P<0.01; **Figure 5**). In both 2021 and 2022, the enzyme activity in the 0–30 cm soil layer for all treatments initially diminished and subsequently grew progressively during the growth period. The alkaline phosphatase activity in the 0–10 cm soil layer was lowest in the Con treatment at every growth stage. In the 10–20 cm soil layer, the T4 treatment exhibited the highest soil alkaline phosphatase activity at each development stage.

### 3.9 Soil urease activity

**Figure 6:**
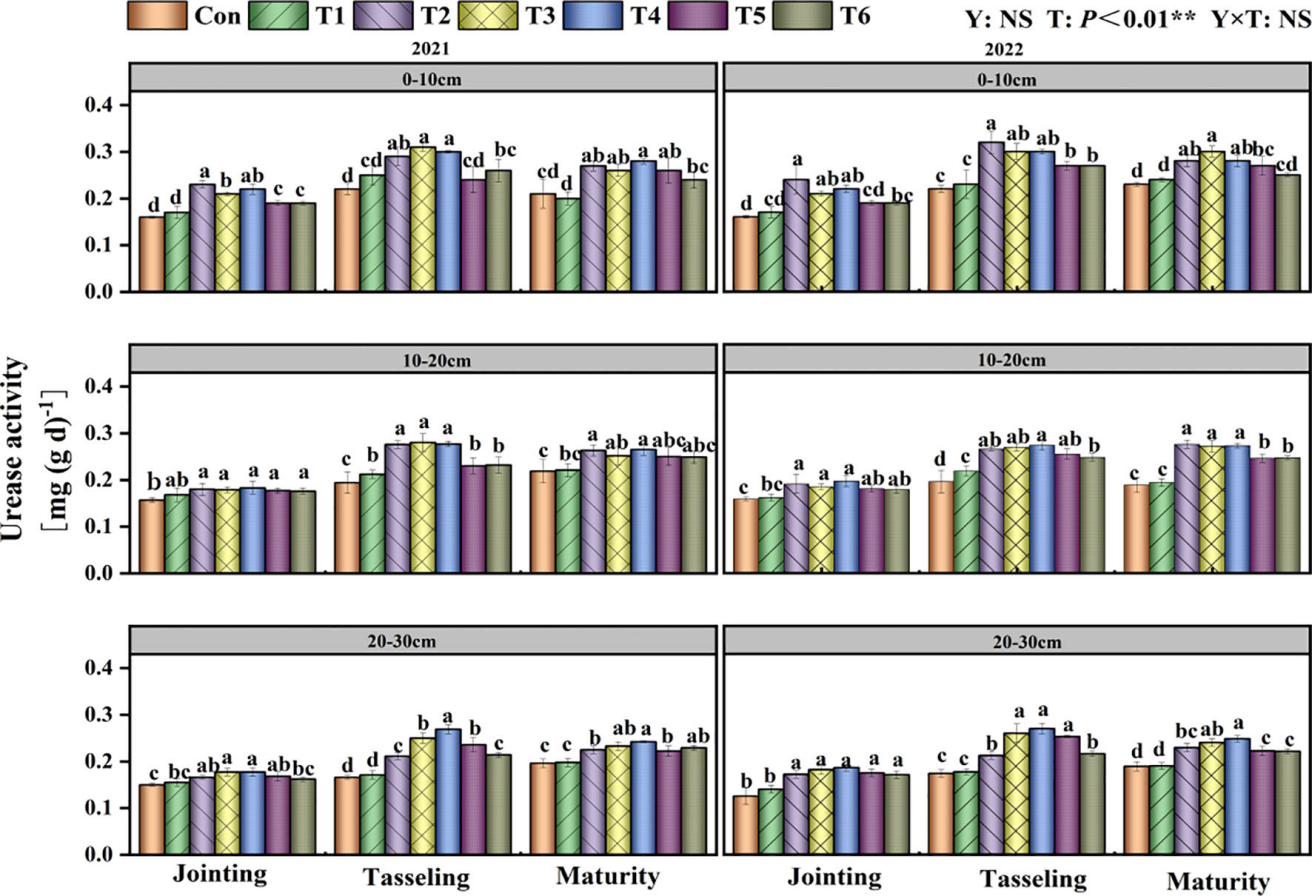
Effects of tillage methods on soil urease activity. Con (rotary ridge tillage), T1 (no-tillage), T2 (raw return + no-tillage), T3 (deep plow straw return + ridge tillage), T4 (deep plow straw return + flat tillage), T5 (raw crushing and return + ridge tillage), and T6 (raw crushing and return + flat tillage).Different alphabets indicate the significance within the same year at 5% level by Duncan' tests. In ANOVA, Y, T represent the variable year, treatment. NS, not significant, (p > 0.05). **, Significant at p < 0.01.

T had a considerable impact on soil urease activity (P<0.01; **Figure 6**). Compared to the T1 treatment, the T2 treatment markedly enhanced soil urease activity in the 0–10 cm soil layer during several growth stages of maize, with increases of 36.29%, 28.40%, and 26.68%, respectively. In the 10–20 cm soil layer, all treatments enhanced soil urease activity relative to the control treatment. In the 20–30 cm soil layer, the soil urease activity in the T3 and T4 treatments was significantly elevated compared to the Con treatment at each maize development stage.

### 3.10 Soil catalase activity

**Figure 7:**
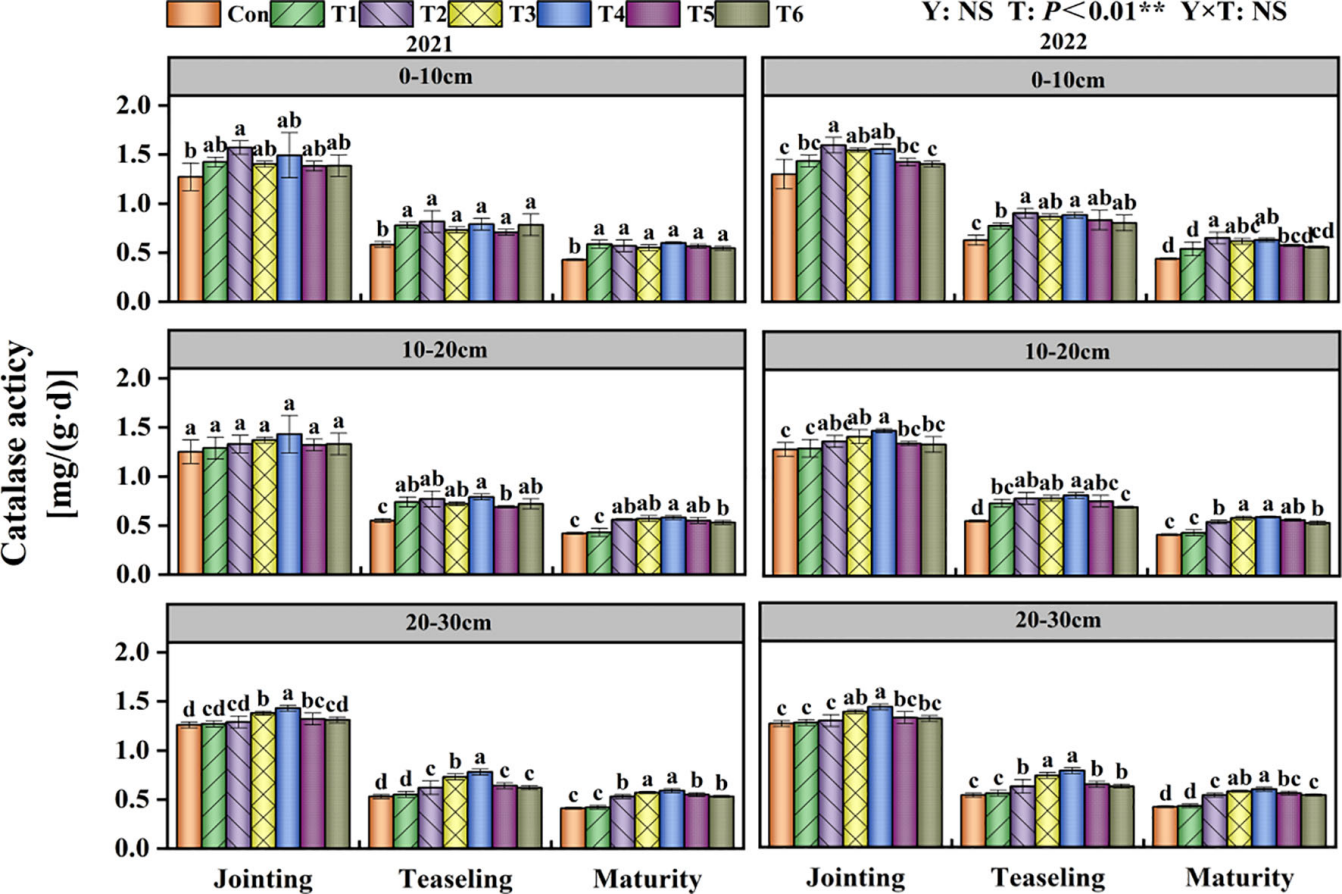
Effects of tillage methods on soil catalase activity. Con (rotary ridge tillage), T1 (no-tillage), T2 (raw return + no-tillage), T3 (deep plow straw return + ridge tillage), T4 (deep plow straw return + flat tillage), T5 (raw crushing and return + ridge tillage), and T6 (raw crushing and return + flat tillage). Different alphabets indicate the significance within the same year at 5% level by Duncan' tests. In ANOVA, Y, T represent the variable year, treatment. NS, not significant, (p > 0.05). **, Significant at p < 0.01.

T had a significant impact on soil catalase activity (P<0.01; **Figure 7**). In the 0–10 cm soil layer, the T2 treatment exhibited significantly higher catalase activity than the Con treatment from jointing to maturity stages, showing increases of 17.76%, 29.13%, and 26.47%, respectively (**Figure 7**). In the 10–20 cm soil layer, the catalase activity in the T4 treatment was the greatest at each development stage. In the 20–30 cm soil layer, the T4 treatment significantly enhanced soil catalase activity throughout the maize growth period compared to the Con treatment.

### 3.11 Maize yield

The impact of various Y and T on 100-Kernels weight and maize yield was highly significant (P< 0.01), and their interaction significantly influenced maize yield (P<0.05) (**Table 5**). In 2021, relative to the Con treatment, the T3 and T4 treatments markedly augmented maize ear length by 10.58% and 9.88%, respectively, diminished bald tip length, and greatly improved 100-Kernels weight by 20.96% and 24.51%, respectively. Furthermore, they markedly enhanced the final yield by 6.00% and 10.44%, respectively. In 2022, the T4 treatment attained the greatest yield of 11130.33 kg·ha^-1^, reflecting substantial increases of 16.81% and 15.69% relative to the Con and T6 treatments, respectively. The enhancement in yield was ascribed to substantial improvements in maize ear length and 100-Kernels weight.

**Table 5:** Effects of tillage methods on yield components of maize.

Year	Treatment	Ear length (cm)	Ear crude (cm)	Bald tip length (cm)	Ear rows	Number per row	100-Kernels weight (g)	Yield (kg hm^-2^)
2021	Con	17.11b	4.94a	2.01ab	16.4ab	36.30a	29.01c	9368.09cd
	T1	18.13ab	4.98a	1.51abc	15.80ab	35.60a	32.08b	9133.25d
	T2	18.39ab	4.98a	0.67cd	16.0ab	35.40a	31.85b	9572.52c
	T3	18.92a	5.04a	1.34bcd	17.20a	39.30a	35.09a	9930.33b
	T4	18.80a	5.06a	0.46d	16.60ab	38.10a	36.12a	10345.77a
	T5	17.81ab	4.97a	0.97cd	16.40ab	35.20a	30.59bc	9420.50cd
	T6	18.87a	5.09a	2.39a	15.6b	37.3a	30.70bc	9376.96cd
2022	Con	17.42b	4.81a	1.96ab	15.40c	35.40a	29.99c	9528.86b
	T1	18.25ab	4.80a	1.29abc	16.20abc	37.0a	32.22b	9536.85b
	T2	19.35a	4.86a	1.04bc	15.80bc	36.10a	33.69b	9745.17b
	T3	19.89a	4.97a	1.09bc	17.6a	38.40a	36.09a	10951.12a
	T4	20.02a	5.18a	0.62c	17.00a	37.40a	37.04a	11130.33a
	T5	17.44b	4.92a	1.32abc	16.00bc	35.60a	31.79bc	9689.00b
	T6	18.65ab	4.98a	2.22a	16.00bc	36.40a	32.08b	9620.51b
Two-way ANOVA	Y	NS	NS	NS	NS	NS	**	**
	T	**	NS	**	*	NS	**	**
	Y×T	NS	NS	NS	NS	NS	NS	*

Con, rotary ridge tillage; T1, no-tillage; T2, straw return + no-tillage; T3, deep plow straw return + ridge tillage; T4, deep plow straw return + flat tillage; T5, straw crushing and return + ridge tillage; T6, straw crushing and return + flat tillage. Different alphabets indicate the significance within the same year at 5% level by Duncan’ tests. In ANOVA, Y and T were variables of year and treatments. ns, not significant, (p > 0.05); *Significant at p< 0.05; **Significant at p< 0.01.

### 3.12 Relationships between the soil indices and maize yield

**Figure 8:**
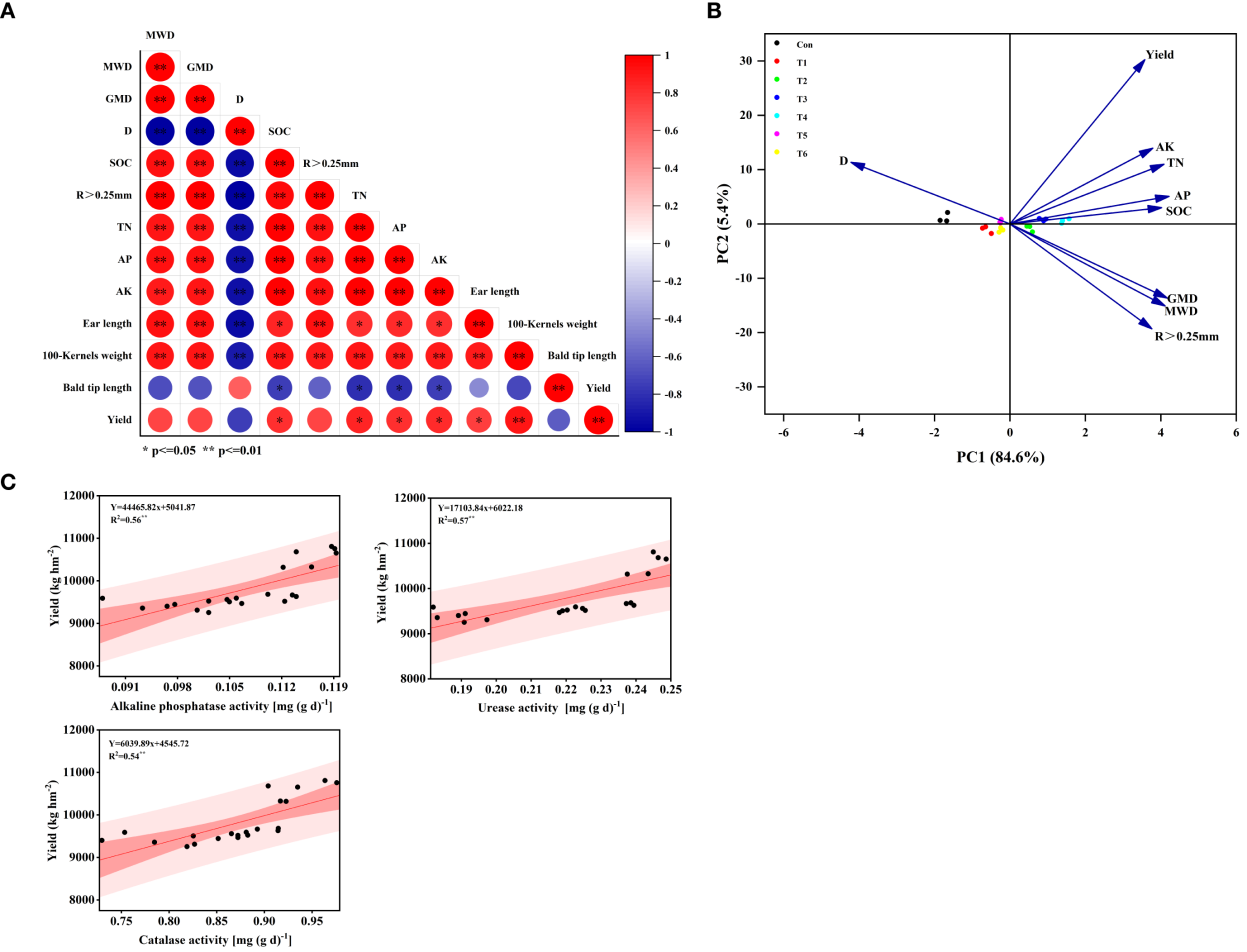
Correlation heatmap **(A)**, principal component analysis **(B)**, and regression analysis **(C)**. Con (rotary ridge tillage), T1 (no-tillage), T2 (raw return + no-tillage), T3 (deep plow straw return + ridge tillage), T4 (deep plow straw return + flat tillage), T5 (raw crushing and return + ridge tillage), and T6 (raw crushing and return + flat tillage). MWD, mean weight diameter; GMD, geometric mean diameter; D, fractal dimension; R>0.25mm, macroaggregate content; TN, total nitrogen; AN, available nitrogen; AP, available phosphorus; AK, available potassium; SOC, soil organic carbon.

The correlation analysis revealed that maize yield was significantly positively correlated with soil SOC, N, P, and K (P<0.05) (**Figure 8A**). Maize yield was significantly positively correlated with 100-Kernels weight and panicle length (*P*<0.01). The MWD, GMD, and R_>0.25 mm_ were significantly positively correlated with the soil nutrient content, 100-grain weight, and ear length (*P*<0.01). These findings indicate that improved soil structural stability could increase the soil nutrient content, thus promoting the transport of nutrients from underground to aboveground parts.

The contribution rates of the first principal component PC1 and the second principal component PC2 were 84.6% and 5.4%, respectively (**Figure 8B**). PC1 was negatively correlated with D and positively correlated with the other indicators. There were significant differences in the T2, T3, T4, and Con treatments along PC1.

There was a significant linear correlation between maize yield and soil alkaline phosphatase activity, urease activity and catalase activity under the different tillage methods (**Figure 8C**) (R^2^ = 0.56, P< 0.05). R^2^ = 0.57, P< 0.05; R^2^ = 0.54, P< 0.05).

## 4 Discussion

### 4.1 Effects of tillage methods on soil water-stable aggregates

The distribution and content of soil aggregates not only influence crop growth and development but also serve as key indicators of soil’s sustainable utilization and erosion resistance capacity (Madari et al., 2004). The conversion between macroaggregates and microaggregates is profoundly affected by tillage methods (Puget et al., 2010). The results demonstrated that all experimental treatments significantly increased the content of water-stable macroaggregates in the 0–10 cm soil layer compared to the Con (**Figure 1**). In the 20–30 cm soil layer, the T4 treatment exhibited the most pronounced increase in macroaggregate content compared to the Con, with significant enhancements in the mean weight diameter MWD and GMD of aggregates by 12.17%-19.27% and 13.40%-18.37%, respectively (**Figure 2**). The study results indicated that straw returning greatly increased the content of water-stable macroaggregates and boosted soil structural characteristics (Li and Zhang, 2019). Furthermore, the two-year results demonstrated that the T4 treatment significantly enhanced the content of water-stable aggregates in both 10–20 cm and 20–30 cm soil layers compared to T2 and T6 treatments. This improvement was accompanied by increased MWD and GMD, reduced fractal dimension (**Supplementary Figure S2**), and elevated organic carbon content in water-stable aggregates (**Figure 2**). This may be attributed to flat planting, which minimizes soil disturbance, hence mitigating structural damage and enhancing soil permeability (West and Post, 2002). After deep plowing with straw returned to the field, the decomposition of crop stubble in deep soil layers increased SOC content and provided essential cementing materials for deep soil particle accumulation (Eynard et al., 2006). This process promotes the formation of large aggregates (Song et al., 2019) and supplies essential energy for crop growth (Yu et al., 2020). The integration of crop residue with conservation tillage has been shown to regulate soil structure, augment soil carbon and nitrogen sequestration, and improve production and soil nutrient levels (Yang et al., 2019; Yuan et al., 2021).

### 4.2 Effects of tillage methods on soil nutrients and enzyme activity

Our study confirmed that straw returning increases soil nutrient content and enzyme activity, consistent with findings reported in previous studies (Liu et al., 2025; Peng et al., 2016; Su et al., 2020). The two-year results demonstrated that compared with Con, the T4 treatment increased SOC content by an average of 15.21% (**Figure 4**), TN content by 26.22% (**Table 2**), AN content by 16.43% (**Table 3**), AP by 26.92% (**Table 4**), and AK by 13.14% (**Table 5**). The 15.21% increase in SOC content under the T4 treatment was statistically significant and ecologically vital. This increment enhanced the soil’s capacity to sequester carbon, thereby mitigating climate change effects through atmospheric CO_2_ reduction (Rattan, 2004; West and Post, 2002). Additionally, higher SOC improved soil structure (Bronick and Lal, 2005), increasing water-holding capacity (Hudson, 1994) - critical for sustaining crop growth during dry periods (Lal, 2020) - and reducing erosion risks. As shown in the correlation analysis, soil aggregate content, MWD, and GMD were significantly positively correlated with soil nutrient content (**Figures 8A, B**). This is attributed to the deep tillage with straw returning to the field improving soil structure. The greater incorporation depth increases the contact area between straw and deep soil, enhancing microbial decomposition. During decomposition, straw significantly increases microbial populations and soil enzyme activity (**Figures 5**-**7**), while activating AP, and AK, thereby improving soil fertility (Piazza et al., 2020). Previous studies have indicated that straw returning can improve soil structure and enhance organic matter input in deeper soil layers (Liu et al., 2021; Zhang et al., 2014). The enhanced soil structure, along with ample organic matter, establishes advantageous nutritional conditions for microbial activity, hence facilitating elevated soil enzyme activity (Ji et al., 2014; Jin et al., 2009).

### 4.3 Correlation analysis of maize yield with soil nutrient levels and enzyme activities

Our findings indicated that between 2021 and 2022, the T4 treatment yielded the maximum maize production (**Table 5**), exhibiting a substantial increase of 10.44% - 16.81% relative to Con. This superior yield performance was primarily attributed to significant increases in both ear length (9.88% - 14.93%) and 100-Kernels weight (23.51% - 24.51%). Correlation and principal component analyses demonstrated strong positive associations between maize yield and both soil aggregate structure and nutrient content (**Figures 8A, B**). Regression analysis revealed a substantial linear correlation between maize yield and soil enzyme activity (**Figure 8C**). Consequently, we ascertain that the integration of tillage systems with straw incorporation enhances the structure of soil water-stable aggregates (Kabiri et al., 2015). Enhanced soil structural stability increases the accumulation of nitrogen, phosphate, and potassium nutrients (Guo et al., 2022), enhances microbial activity (Zheng et al., 2022), and rises enzyme activity (Costa et al., 2024). These enhancements promote nutrient transfer from leaves to grains (Tang et al., 2018), augmenting both grain nutrient content and 100-Kernels weight, celevating maize output. These results demonstrated that deep plowing straw returning + flat tillage enhanced soil health and increased maize yield, supporting sustainable agriculture in the black soil region. This approach provided a viable strategy to balance productivity with long-term soil fertility - a key principle for preserving this vulnerable ecosystem. For farmers, deep plowing straw returning+ flat tillage boosted yields and income while maintaining soil quality. For policymakers, promoting this practice through technical training and incentives facilitated sustainable black soil management, safeguarding future agricultural productivity.

### 4.4 Limitations and future research

This study was conducted over a two-year period. To further validate these findings, the research team plans to continue long-term monitoring in the region. The present investigation was confined to a singular agroecological zone. Our team did not investigate ecological areas with differing climatic conditions. To thoroughly assess the impact of farming practices in various environments on maize production, soil structure, and fertility. Our team plans to examine the response effects of diverse tillage techniques across various ecological zones in the future, thereby offering significant theoretical insights for the progression of sustainable agriculture and field management in this area.

## 5 Conclusion

This two-year study illustrates that deep plowing straw returning + flat tillage markedly enhances soil structure in the semi-arid districts of western Heilongjiang Province, thereby establishing it as an efficacious tillage management strategy. The results indicated that, in comparison to conventional tillage, this tillage method enhanced soil aggregate structure, elevated soil organic carbon, total nitrogen, available nutrients, and enzyme activities, increased the 100-Kernels weight of maize, and ultimately augmented maize yield by 10.44% to 16.81%. These findings provide valuable practical guidance for local policymakers and farmers in the study region.

## Data Availability

The original contributions presented in the study are included in the article/**Supplementary Material**. Further inquiries can be directed to the corresponding authors.
