# Supplementary material for: Optimize the farming system to improve the physical and chemical properties of soil in Northeast China, thereby increasing maize yield
Source: Front Plant Sci. 2025 Aug 11;16:1626882. doi: 10.3389/fpls.2025.1626882 (PMC12375559; doi:10.3389/fpls.2025.1626882)
Supplement: Supplementary file 1 [file Table1.docx]

Table S1.

Effects of tillage methods on soil available nitrogen content.

| Soil depth（cm） | Treatments | Soil available nitrogen content（mg/kg） | | | | | |
| --- | --- | --- | --- | --- | --- | --- | --- |
|  |  | 2021 | | | 2022 | | |
|  |  | Jointing | Tasseling | Maturity | Jointing | Tasseling | Maturity |
| 0~10 | Con | 116.90c | 101.03d | 116.90b | 118.30d | 103.83c | 117.37b |
|  | T1 | 122.50bc | 109.43bc | 126.70ab | 123.43c | 112.70bc | 127.63abc |
|  | T2 | 129.97a | 117.83a | 137.90a | 131.83a | 123.90a | 139.30a |
|  | T3 | 122.03bc | 110.83bc | 127.63ab | 125.77bc | 120.63ab | 131.37ab |
|  | T4 | 127.17ab | 115.50ab | 131.83a | 129.97ab | 122.7a | 135.57ab |
|  | T5 | 121.57bc | 106.17cd | 125.30ab | 122.50cd | 114.57ab | 129.03abc |
|  | T6 | 120.63bc | 102.43d | 123.90ab | 121.10cd | 111.30bc | 125.30bc |
| 10~20 | Con | 98.23b | 97.30c | 110.37d | 103.97d | 99.63c | 112.47c |
|  | T1 | 106.17b | 103.37bc | 115.97c | 109.90c | 104.30bc | 117.83bc |
|  | T2 | 116.43a | 108.97ab | 121.10bc | 117.37b | 111.77a | 122.03abc |
|  | T3 | 120.63a | 110.83ab | 124.37ab | 122.50ab | 114.57a | 128.10ab |
|  | T4 | 124.37a | 114.57a | 129.10a | 126.70a | 115.97a | 132.77a |
|  | T5 | 118.77a | 104.30bc | 122.97ab | 119.70b | 106.63b | 125.30ab |
|  | T6 | 105.70b | 100.57c | 119.23bc | 110.37c | 105.70b | 120.40abc |
| 20~30 | Con | 90.30b | 85.17d | 103.37c | 92.17d | 90.77c | 104.77c |
|  | T1 | 91.70b | 87.03cd | 104.30c | 95.20cd | 92.17bc | 106.63c |
|  | T2 | 96.83ab | 89.93bcd | 108.03bc | 97.30cd | 93.57bc | 108.50c |
|  | T3 | 103.37a | 95.43ab | 121.57a | 106.17ab | 102.90a | 122.50a |
|  | T4 | 104.77a | 98.70a | 123.90a | 107.57a | 103.37a | 124.37a |
|  | T5 | 98.70ab | 93.10ab | 116.43ab | 100.10bc | 97.30b | 119.23ab |
|  | T6 | 97.77ab | 91.23bc | 109.43bc | 98.23cd | 94.50bc | 112.23bc |

Note: Con (Rotary ridge tillage), T1 (No-tillage), T2 (Straw returning + no-tillage), T3 (Deep plowing straw returning + ridge tillage), T4 (Deep plowing straw returning + flat tillage), T5 (Straw crushing and returning + ridge tillage), T6 (Straw crushing and returning + flat tillage). Different small letters above the bars indicate significant differences between treatments (p<0.05).
