# Supplementary figures and images for: Optimize the farming system to improve the physical and chemical properties of soil in Northeast China, thereby increasing maize yield
Source: Front Plant Sci. 2025 Aug 11;16:1626882. doi: 10.3389/fpls.2025.1626882 (PMC12375559; doi:10.3389/fpls.2025.1626882)

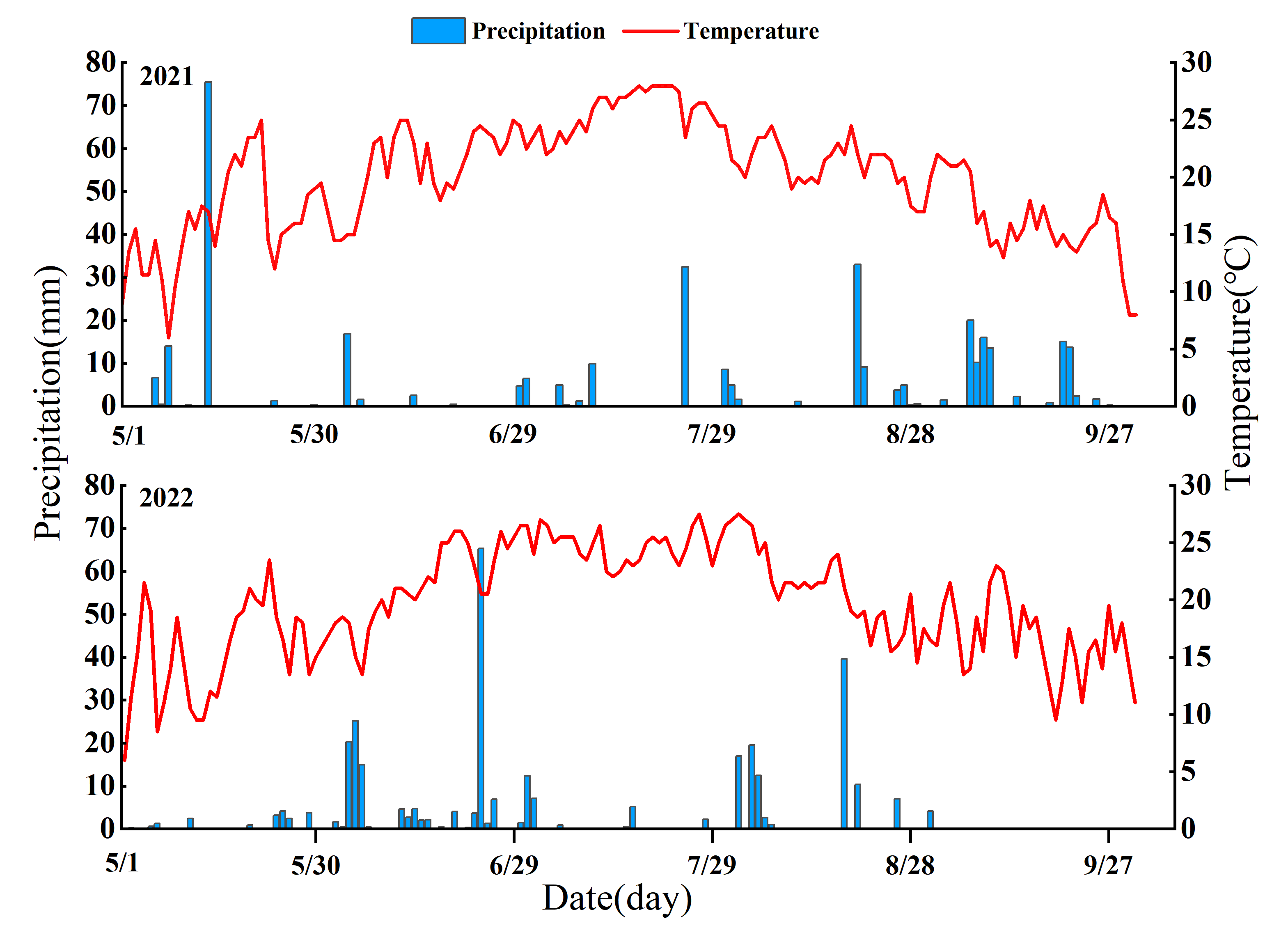

Supplement: Supplementary file 2 [file Image1.tif]

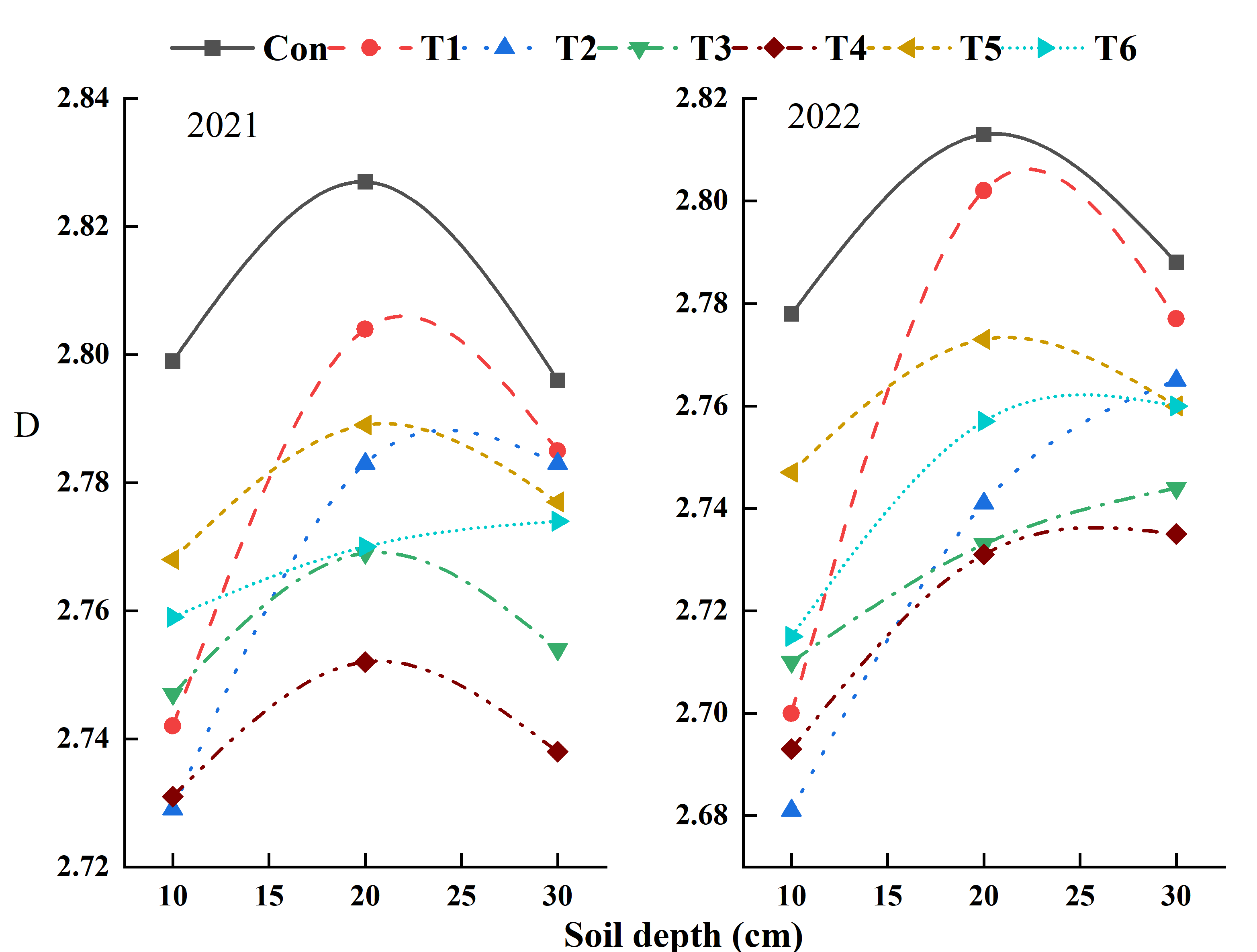

Supplement: Supplementary file 3 [file Image2.tif]

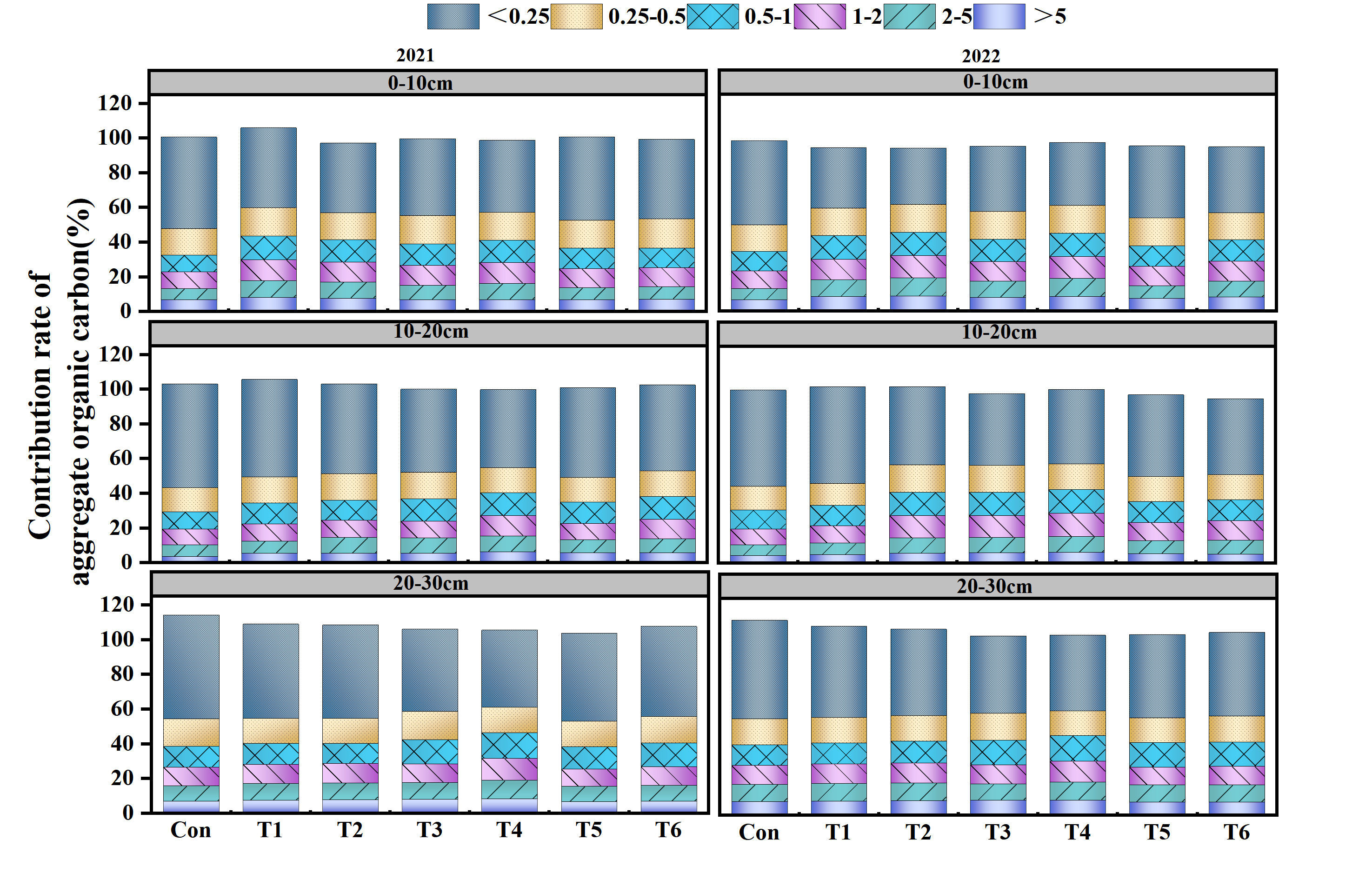

Supplement: Supplementary file 4 [file Image3.tif]
